# Editorial Comment: Sexual Function Outcomes After Surgical Treatment of Penile Fracture

**DOI:** 10.1590/S1677-5538.IBJU.2022.02.03

**Published:** 2022-03-01

**Authors:** Rodrigo Barros de Castro

**Affiliations:** 1 Universidade Federal Fluminense Hospital Universitário Antônio Pedro Serviço de Urologia Niterói RJ Brasil Serviço de Urologia, Hospital Universitário Antônio Pedro - Universidade Federal Fluminense - UFF, Niterói, RJ, Brasil

Yassine Ouanes 1, Mohamed Hafedh Saadi 2, Houssem Haj Alouene 1, Mokhtar Bibi 1, Ahmed Sellami 1, Sami Ben Rhouma 1, Yassine Nouira 1

Sex Med. 2021 Jun;9(3):100353.

## COMMENT

Evidence has shown a trend towards urgent surgical repair of penile fracture (PF) in order to have more adequate functional and cosmetic results in relation to conservative treatment (1). The surgery aims to restore the anatomical and functional integrity of the penis, to avoid complications such as penile curvature, erectile dysfunction (ED), penile plaque and painful erection (2).

In this recent publication, the authors evaluated 138 patients with PF over 19 years to identify the factors that can influence the sexual function after surgical repair. Clinical features, perioperative findings, time from injury to surgery, lesions of the corpora cavernosa and presence of urethral injury were reviewed. Sexual function was evaluated six months after surgical repair by applying the International Index of Erectile Function-5 (IIEF-5) questionnaire and assessing penile curvature and the presence of a painful intercourse. They used elective incision in most cases (81.2%), while subcoronal degloving incision was used only in cases when it was impossible to locate the tunical tear by physical examination.

The authors found ED in 24 cases (17.4%), penile curvature that was interfering with sexual intercourse in 21 patients (15.3%), and painful intercourse in 18 patients (13%). The presentation delay varied from 1 hour to 5 days (mean = 16.8 hours). They discovered that presentation time delay, tunical leak located in the proximal shaft of the penis and elective incision were the three factors associated with higher penile curvature. They also found that presentation delay, the injury in the proximal shaft and elective incision were the most related factors to ED. They concluded that elective incision should be performed for tunical leaks located only in the distal shaft of the penis, while for those located in the proximal shaft, circumferential degloving incision should be considered to reduce the risk of penile curvature caused by this type of lesion.

There are many studies (3–5) demonstrating safety and efficacy of the surgical treatment of PF and the positive impact on sexual function. The majority of surgeons use subcoronal degloving incision with low rates of surgical wound complications (3,6). However, some authors advocate use of a small skin incision to access the exact point of injury, to avoid the complications of degloving and postectomy (7, 8). Finally, regardless of incision type, immediate surgical repair of PF is the gold standard approach and should be strongly recommended to avoid long-term complications, especially penile tortuosity and ED. It is noteworthy that erectile function is a complex phenomenon comprising emotional, physical and relational aspects and can be affected after surgical treatment of PF.
